# A technical appraisal of guidelines for the management of skin rash in patients on chemotherapy and targeted therapy

**DOI:** 10.1186/s12913-019-4539-6

**Published:** 2019-10-16

**Authors:** Fangyuan Zhang, Sumei Lv, Yating Feng, Xuan Yang, Wanmin Qiang

**Affiliations:** 1 Tianjin Medical University Cancer Institute and Hospital, National Clinical Research Center for Cancer, Hexi District, 1 West Lake Road, Tianjin, CN China; 2 Forth Hospital of Hebei Medical University, Shijiazhuang, Hebei China; 3 The First Affiliated Hospital of Zhengzhou University, Zhengzhou, Henan China

**Keywords:** Chemotherapy, Targeted therapy, Rash, Guidelines, AGREE

## Abstract

**Background:**

Skin rash remains one of the most prevalent and troublesome clinical problems experienced by patients on chemotherapy and targeted therapy. To ensure high-quality care, guidelines are seen as the best guidance. Considering the quality of guidelines varies greatly, a systematical appraisal of the methodological quality of guidelines for the management of skin rash in patients on chemotherapeutic drugs and targeted anticancer therapies was undertaken, in order to identify appropriate ones for healthcare professionals.

**Methods:**

A systematic search of databases and Internet was conducted to obtain pertinent guidelines. Two reviewers independently assessed the eligibility of guidelines according to the inclusion criteria. Then the guidelines included were appraised by three researchers with the methodological quality of eligible guideline using *Appraisal of Guidelines for Research and Evaluation II* (AGREEII).

**Results:**

Totally nineteen guidelines met the inclusion criteria. The quality ranged from good to acceptable in scope and purpose (mean: 78.80%, range: 66.67–94.44%) and clarity of presentation domains (mean: 85.38%, 75.00–91.67%), but not in stakeholder involvement (mean: 50.15%, range: 36.11–75.00%), rigor of development (mean: 23.65%, range: 6.25–70.83%), applicability (mean: 23.96%, range: 4.17–52.08%), and editorial independence domains (mean: 45.18%, range: 0.00–87.50%). Overall, two guidelines were classified as “recommended”.

**Conclusions:**

Only two guidelines were recommended to manage skin rash in patients on chemotherapy and targeted therapies, most guidelines issued were of low to moderate quality. Thus, more attention should be paid to the methodological quality of guideline development in this field.

## Background

More than one hundred kinds of drugs are widely used in the clinical treatment of different cancers, and they can be divided into non-targeted agents and targeted agents [1]. Although conventional chemotherapy remains an essential mainstay of cancer treatment, targeted drugs are increasingly being applied to treat cancer because of better tolerance. Due to the disturbance of specific cell cycle phases and target molecules are present in the skin, skin reactions are common side effects of many classic chemotherapeutic agents and the newer molecular targeted therapies [2–4]. The incidence of skin reactions varies in cancer patients according to the chemotherapeutic agents used, and would increase when together with targeted therapies [5, 6]. The cutaneous adverse events of conventional chemotherapy and targeted therapies could have a negative impact on patients’ physical, psychological and social well-being, and frequently cause dose reduction and delay, or even discontinuation of treatment [7–9]. Skin rash acts as one of the most common dermatological toxicities, appropriate management strategies are necessary to improve health-related quality of life and outcomes of patients on chemotherapeutic drugs and targeted anticancer therapies [1].

Clinical practice guidelines (‘guidelines’) are defined as the systematically developed statements to assist practitioner and patient decisions about appropriate health care for specific clinical circumstances by the Institute of Medicine (IOM) [10]. Many scientific societies and specialist groups have developed and issued guidelines for the management of skin rash in patients on chemotherapy and targeted therapy, in order to rationalize and standardize the clinical practice. However, the value of guidelines is proportional to the quality of the guidelines, flawed guidelines may result in the promotion of ineffective, or even harmful practices to patients, and a waste of limited healthcare resources [11, 12]. Efforts are greatly desired to evaluate the methodological quality of guidelines before application to clinical practice. Thus, we conducted this study to appraise the methodological quality of guidelines for the management of skin rash in patients on chemotherapy and targeted therapy, and to identify appropriate guidelines for healthcare professionals to provide better quality care for patients.

## Methods

### Electronic database searches

A systematic literature search was performed. PubMed and Embase were searched to identify all possible guidelines. Articles published in English between the inception of each database and October 2018, were searched for controlled vocabulary terms specific to each database related to neoplasms, skin toxicity, rash, guidelines. Detailed search strategies were provided in Supplementary Methods (Additional file 1) [13]. We also manually reviewed the references of the included studies.

### Internet searches

Besides, a through internet search was conducted to identify pertinent guidelines from the website of the international cancer organizations and guideline clearinghouses. The guideline resource section in each website was carefully reviewed or searched, and any relevant guidelines were included. A list of these organizations and clearinghouses was shown in Supplementary Methods (Additional file 2).

### Eligibility criteria

We included guidelines according to the following criteria: (1) Target population: Adults patients with cancer, there were no restrictions on type, stage, or site of cancer; (2) Scope: Management strategies of skin rash in patients on chemotherapeutic drugs and targeted therapy, included prophylaxis, assessment, pharmaceutical or non-pharmaceutical treatment; (3) Development method: Guidelines were developed based on evidence, consensus and/or expert opinion; (4) Development organization: Guidelines were developed by regional, national or international professional organization or societies, or by a national or international expert panel; (5) Form: Full texts available; (6) Others: If there had updated versions, only the latest one was included. Protocol, interpretation and translation of guidelines were excluded.

### Guideline selection

After removing duplicate records, two researchers (YT, YX) independently assessed the eligibility of all guidelines. Disagreements regarding inclusion in the final review were resolved through discussion and consensus. A third researcher (WM) was consulted if disagreement cannot be resolved between the two researchers. Besides, the guidelines included were classified into two types: evidence-based guidelines (EBGs) and consensus-based guidelines (CBGs) [14]. If a guideline reported a search strategy, the quality of evidence on which a recommendation is based and grading of recommendation, then this guideline is judged as EBG. CBG is defined as a document representing the collective opinion of an expert panel without illustrating the source of evidence or grading of recommendation.

### Quality appraisal

The Appraisal of Guidelines for Research and Evaluation II (AGREEII) tool was used to critique the guidelines. AGREEII is a guideline quality appraisal tool with high construct validity, which consists of 23 items arranged into 6 domains: scope and purpose (3 items), stakeholder involvement (3 items), rigor of development (8 items), clarity of presentation (3 items), applicability (4 items), and editorial independence (2 items) [15, 16]. Each item is scored between strongly agree (7) and strongly disagree (1). The items scores within a domain were then added and calculated as a percentage. A domain was determined to be effectively addressed if its score was≥60% [17–20]. All members of the research team undertook a training review process to ensure consistency and reliability in grading. Assessment of all the included guidelines was performed independently by three researchers (FY, SM and WM). Prior to the formal assessment, we conducted a pre-assessment by randomly choosing five guidelines. The intra-class correlation coefficients (ICCs) were calculated to assess the intra-rater reliability of the three appraisers. Only when ICC was more than 0.80, the formal assessment would start.

Overall guideline assessment reached consensus according to the quality of the guideline. Each guideline was classified as “recommended”, “recommended with modifications” or “not recommended”.

## Results

### Guidelines included

**Fig. 1:**
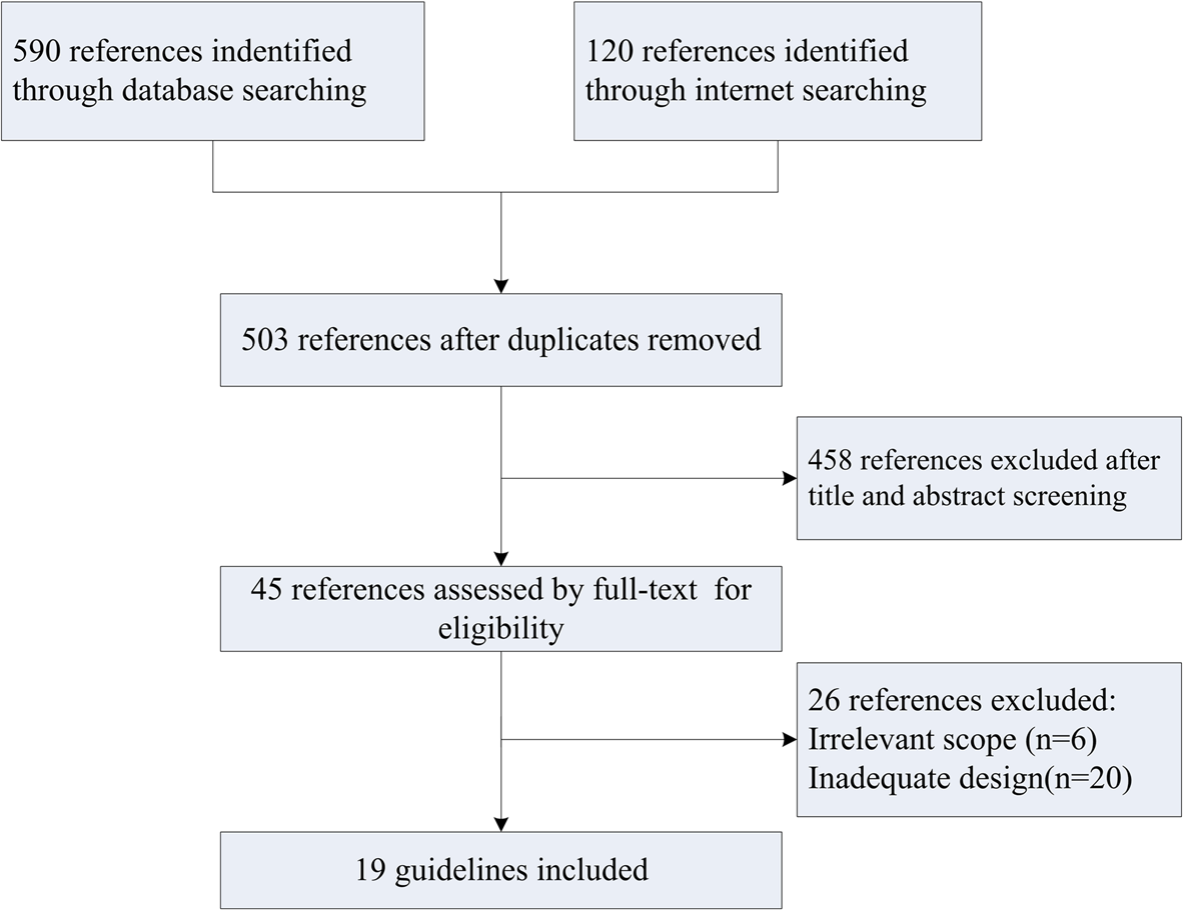
Flow chart of the systematic review selection procedure

A total of 710 references were identified from electronic databases, international cancer organizations and guideline clearinghouses. Of these, 458 were excluded by screening the title and abstract, and 26 were excluded by reviewing the full texts of potentially eligible articles. Finally, 19 guidelines were included in this review (Fig. 1) [21–39]. The characteristics of the included guidelines are presented in Table 1. All guidelines included were developed by an interdisciplinary expert panel, in which there were five guidelines issued by specific society or organization focused on adverse events caused by chemotherapy or targeted therapy. As for the methodology of guideline development, only two guidelines were judged as EBG.

**Table 1:** Characteristics of guidelines included in this study

Title	Year of publication	Country/region	Level of development	Organization	Authors number	Number of references	Type of guideline
EGFR inhibitor-associated cutaneous toxicities: An Evolving Paradigm in Clinical Management [21]	2007	USA	International	Not specified	6	58	CBG
Clinical significance and treatment of skin rash from erlotinib in NSCLC patients: results of an Experts Panel Meeting [22]	2008	Italy	National	Not specified	15	37	CBG
An interdisciplinay consensus on managing skin reactions associated with EGFRI [23]	2008	USA	National	Not specified	3	31	CBG
Expert consensus on the management of erlotinib-associated cutaneous toxicity in the UK [24]	2009	UK	National	Not specified	9	28	CBG
Sunitinib in metastatic renal cell carcinoma: recommendations for management of noncardiovascular toxicities [25]	2011	Canada, USA France,	International	Not specified	16	55	CBG
Management of cutaneous side effects of EGFR inhibitors: recommendation from a German expert panel [26]	2011	Germany	National	Not specified	12	36	CBG
Clinical practice guideline for the prevention and treatment of EGFR inhibitor-associated dermatologic toxicities [27]	2011	France, Italy, USA,Singapore	International	Skin toxicity study group (STSG)	7	114	EBG
Interdisciplinary management of EGFR-inhibitor- induced skin reactions: a German expert opinion [28]	2011	Germany	National	Not specified	12	81	CBG
Management of skin toxicity associated with cetuximab treatment in combination with chemotherapy or radiotherapy [29]	2011	Italy	National	Not specified	7	68	CBG
Management of cutaneous adverse events induced by anti-EGFR: a French interdisciplinary therapeutic algorithm [30]	2012	France	National	Not specified	21	32	CBG
Clinical practice guideline for the prevention and treatment of rash in patients treated with EGFR inhibitor therapies [31]	2012	Canada	Regional	TGHNTT	Not specified	73	CBG
Daily baseline skin care in the prevention, treatment, and supportive care of skin toxicity in oncology patients: recommendation from a multinational expert panel [32]	2013	Europe	International	No specified	7	48	CBG
Management of the adverse events of afatinib: a consensus of the recommendations from the Spanish expert panel [33]	2015	Spain	National	No specified	8	34	CBG
Expert Consensus on the Management of Adverse Events from EGFR Tyrosine Kinase Inhibitors in the UK [34]	2015	UK	National	No specified	13	46	CBG
Practical recommendations for rash and diarrhea management in Indian patients treated with TKI for the treatment of NSCLC [35]	2016	India	National	No specified	11	7	CBG
Management of skin reactions during cetuximab treatment in association with chemotherapy or radiotherapy: update of the Italian expert recommendations [36]	2016	Italy	National	No specified	7	53	CBG
BRAF/MEK Inhibitor Therapy: Consensus Statement on Managing Adverse Events and Potential Drug Interactions [37]	2017	USA	National	The Melanoma Nursing Initiative	2	33	CBG
Prevention and management of epidermal growth factor receptor tyrosine kinase inhibitor-related skin toxicities [38]	2017	China	Regional	TDA	6	33	CBG
Putting evidence into practice: skin reactions [39]	2017	USA	National	ONS	10	98	EBG

*Abbreviations*: *EGFR* epidermal growth factor receptor, *ESMO* European society for medical oncology, *NSCLC* non-small cell lung cancer, *TDA* Taiwanese Dermatological Association, *TGHNTT* thoracic malignancies, gastrointestinal, head and neck tumour teams, *TKIs* tyrosine kinase inhibitors, *ONS* oncology nursing society

### Quality appraisal

#### Overall quality

Table 2 shows the standardized domain scores of each included guideline and their overall assessment. The quality of guidelines varied greatly, from fulfilling most of the AGREEII criteria to fulfilling only two. Among six domains, only two domains of “scope and purpose” and “clarity and presentation” scored over 60%. Overall, two guidelines (10.53%) were classified as “recommended”, ten guidelines (52.63%) were “recommended with modification”, while the rest (36.84%) were “not recommended”.

**Table 2:** Standardized domain scores (%) and overall assessment (*N* = 19)

Guidelines	Scope and purpose	Stakeholder involvement	Rigor of development	Clarity of presentation	Applicability	Editorial independence	Overall assessment
USA 2007 [21]	77.78	50.00	7.29	80.56	24.75	29.17	Not recommended
Italy 2008 [22]	75.00	47.22	6.25	88.89	23.33	8.33	Not recommended
USA 2008 [23]	80.56	52.78	9.38	83.33	14.58	29.17	Not recommended
UK 2009 [24]	80.56	75.00	17.71	91.67	25.42	20.83	Not recommended
International 2011 [25]	69.44	50.00	19.79	80.56	29.17	70.83	Recommended with modifications
Germany 2011 [26]	69.44	41.67	6.25	75.00	10.42	25.00	Not recommended
STSG 2011 [27]	69.44	52.78	61.67	91.67	52.08	66.67	Recommended
Germany 2011 [28]	66.67	44.44	25.00	88.89	20.83	87.50	Recommended with modifications
Italy 2011 [29]	80.56	50.00	19.58	88.89	20.83	20.83	Not recommended
France 2012 [30]	86.11	50.00	52.08	83.33	22.92	0.00	Recommended with modifications
Canada 2012 [31]	83.33	55.56	45.83	80.56	35.42	83.33	Recommended with modifications
Europe 2013 [32]	83.33	52.78	25.00	77.78	21.25	58.33	Recommended with modifications
Spain 2015 [33]	83.33	58.33	12.50	88.89	25.50	50.00	Recommended with modifications
UK 2015 [34]	80.56	50.00	17.71	91.67	25.42	66.67	Recommended with modifications
India 2016 [35]	83.33	36.11	8.33	88.89	21.67	54.17	Recommended with modifications
Italy 2016 [36]	80.56	50.00	25.42	86.11	22.92	0.00	Not recommended
USA 2017 [37]	83.33	36.11	8.33	88.89	21.67	54.17	Recommended with modifications
China 2017 [38]	69.44	50.00	10.42	77.78	4.17	75.00	Recommended with modifications
ONS 2017 [39]	94.44	50.00	70.83	88.89	32.92	58.33	Recommended
Median	78.80	50.15	23.65	85.38	23.96	45.18	

##### Scope and purpose

The median score for the scope and purpose domain was 78.80% (range: 66.67–94.44%). Most guidelines clearly described overall objectives, health questions and target populations.

##### Stakeholder involvement

The median score for the stakeholder involvement domain was 50.15% (range: 36.11–75.00%). Only the UK 2009 guideline scored above 60% [24]. No guidelines clearly described their numbers’ roles in the guideline development process. Besides, methodology experts and economists were not included in any guidelines. Only one guideline reported consideration of the views and preferences of patient representative (UK 2009) [24].

##### Rigor of development

The median score for the rigor of development domain was 23.65% (range: 6.25–70.83%). Only STSG 2011 and ONS 2017 scored over 60%, as they used systematic methods of searching for evidence and for formulating recommendations [27, 39]. Only Canada 2012 clearly described methods for conducting external reviews [31]; only ONS 2017 described their procedures for updating guidelines [39].

##### Clarity of presentation

The median score in this domain was 85.38% (range: 75.00–91.67%), with all guidelines scoring over 60%. All of the guidelines included could provide specific, unambiguous and easily identifiable recommendations.

##### Applicability

The median score for the applicability domain was 23.96% (range: 4.17–52.08%), with no guideline scoring over 60%. Almost all of the guidelines failed to describe the facilitators and barriers of their applications and did not sufficiently consider the costs of applying their recommendations.

##### Editorial Independence

The median score for the editorial independence domain was 45.18% (range: 0.00–87.50%), with six guidelines scoring above 60%. Most guidelines failed to report a statement of “the views or interests of the funding body have not influenced the final consensus or recommendations” or a “no funding” statement.

## Discussions

### Characteristics of included guidelines

The first guideline on the management of skin rash in patients on chemotherapy and targeted therapy was published in 2007. Since than, the number had grown rapidly, up to 19 guidelines in 2018. However, lots of guidelines were judged as CBG, as their recommedations were formed by expert opinion or literature review, but did not provide rating of both the quality of the evidence and strength of the recommendations, which made them less trustworthy. Thus, in order to ensure that guidelines are of a high methodological quality, it is essential to follow a evidence-based guideline development standard, such as the Grading of Recommendations Assessment, Development and Evaluation (GRADE) [40].

### Quality of the guidelines

Of 19 guidelines included, moderate to high scores were achieved in domains of “clarity of presentation”, “scope and purpose”, and “stakeholder involvement”. Mean scores for domian of “applicability” were the lowest, showed that a gap currently exists between the evidence provided and its applicability in the clinical setting, and was in contrast with the need for clarity and user friendliness advocated by some authors [41–43]. Regarding the domain of “rigor of development” with the second lowest mean scores, most guidelines were not based on a systematic review of the literature and were lack of grading of the level of evidence and recommendations, and did not provide recommendations explicitly linked to evidence, which would lower target users’ confidence [17]. Information on “editorial independence”, the most common source of bias in guideline development, was particularly important, but was neglected in most guidelines, which might be associated with differences in recommendations [44, 45]. As a result, only two guidelines met the criteria of AGREEII and were ranked as “recommended”, which meaned that the rest were of a great room to improve the methodological quality, and we should be cautious when application.

### Suggestions to improve guideline’s quality

First of all, professional organizations or socities at national or international level should take responsibility and produce fewer but more trustworthy guidelines based on evidence, in order to avoid a potential waste of scarce guidelines development resources [40]. Then, a panel of multidisciplinary experts should be founded, especially methodology experts shold be included. The most important part is that developers should comply with the definition of guideline by IOM and evidence-based guideline development standard, such as standards from SIGN and NICE [46]. A critcial appraisal of guideline using AGREEII should be considered before release, to make sure if quality standards are met. Furthermore, journal editors ought to set higher standards for peer review, only those guidelines of high quality could be considered for publication [44]. What is more, developers need to update guidelines regularly, and the process should follow the standard of the Guidelines International Network Updating Guidelines Working Group, as it could minimize the risk of bias when update [47].

### Strengths and limitations

This is the first study to systematically review all available guidelines of the management of skin rash in patients on chemotherapy and targeted therapy, with the aim to screen guidelines with high quality, and provide healthcare professionals with evidence-based rocommendations to manage skin rash. We have performed a comprehensive search to identify relevent guidelines, and adopted well-accepted AGREEII to appraise the methodological quality and derive overall assessment of the guidelines.

Although AGREEII appears to be the best methodological tool available, it does not consider the relative importance of six domains of quality. This suggests that the domains of AGREEII should not be weighed equally, such as the domain of “rigor of development” should be of more weight [17, 48]. Besides, the AGREE instrument is developed both for quality assessment and report [49]. Especially for the domain of “editorial independence”, we would consider that low scores in this domain may not reflect a real influence of the funding body in the guidelines development process, but rather reflect an insufficient or a not very explicit reporting of potential conflicts of interest. However, it is impossible to find if the authors chose not to disclose such conflicts [14]. Moreover, guidelines only in English were included, eligible guidelines in other languages were possible missed.

## Conclusions

Only two guidelines were recommended to manage skin rash in patients on chemotherapy and targeted therapy, most guidelines issued were of low to moderate quality. More attention should be paid on the methodological quality of guideline development in this field, particularly in the domains of “rigor of development”, “applicability”, and “editorial independence”.

## Supplementary information

**Additional file 1.** Search method.

**Additional file 2.** Internet list.

## Data Availability

All data generated or analysed during this study are included in this published article.

## Abbreviations

AGREE: Appraisal of guidelines for research and evaluation tool
CBE: Consensus-based guideline
CRR: Chemotherapy related rash
EBG: Evidence-based guideline
EGFR: Epidermal growth factor receptor
ESMO: European society for medical oncology
NSCLC: Non-small cell lung cancer
ONS: Oncology nursing society
TDA: Taiwanese Dermatological Association
TGHNTT: Thoracic malignancies, gastrointestinal, head and neck tumour teams
TKIs: Tyrosine kinase inhibitors
