# Supplementary material for: A technical appraisal of guidelines for the management of skin rash in patients on chemotherapy and targeted therapy
Source: BMC Health Serv Res. 2019 Oct 16;19:704. doi: 10.1186/s12913-019-4539-6 (PMC6794896; doi:10.1186/s12913-019-4539-6)
Supplement: Supplementary file 1 — Additional file 1. Search method. [file 12913_2019_4539_MOESM1_ESM.docx]

**Additional file 1: Search Strategy**

| **Datebase** | **Search strategy** |
| --- | --- |
| Pubmed | #1 neoplasms[mesh]  #2 exanthema[mesh]  #3 practice guideline[publication type]  #4 neoplasia*[title/abstract] or tumor*[title/abstract] or malignanc*[title/abstract] or cancer[title/abstract] or carcinoma*[title/abstract]  #5 skin reaction*[title/abstract] or cutaneous reaction*[title/abstract] or dermtol* reaction[title/abstract] or skin toxicit*[title/abstract] or cutaneous toxicit*[title/abstract] or dermatol* toxicity[title/abstract] or rash[title/abstract] or exanthem[title/abstract]  #6 guideline[publication type] or recommendation*[title/abstract] or standard*[title/abstract] or consensus*[title/abstract] or guideline*[title/abstract]  #7 #1 or #4  #8 #2 or #5  #9 #3 or #6  #10 #7 and #8 and #9 |
| Embase | #1 malignant neoplasm[emtree]  #2 rash[emtree]  #3 practice guideline[emtree]  #4 cancer*[title/keyword] or malignant neoplasm*[title/keyword] or malignant neoplastic disease* [title/keyword] or malignant tumor[title/keyword] or malignant tumour[title/keyword] or neoplasia, malignant[keyword] or tumor, malignant[keyword] or tumour, malignant[keyword]  #5 exanthem[title/keyword] or rash[title/keyword] or exanthema[title/keyword] or skin eruption[title/keyword] or skin rash[title/keyword]  #6 skin reaction*[title/keyword] or skin side reaction*[title/keyword] or skin toxicit*[title/keyword] or cutaneous reaction*[title/keyword] or cutaneous side reaction*[title/keyword] or cutaneous toxicit*[title/keyword]  #7 guideline*[title/abstract] or recommendation*[title/abstract] or standard*[title/abstract] or consensus* [title/abstract]  #8 #1 or #4  #9 #2 or #5 or #6  #10 #3 or #7  #11 #8 and #9 and #10 |
