# Supplementary material for: A technical appraisal of guidelines for the management of skin rash in patients on chemotherapy and targeted therapy
Source: BMC Health Serv Res. 2019 Oct 16;19:704. doi: 10.1186/s12913-019-4539-6 (PMC6794896; doi:10.1186/s12913-019-4539-6)
Supplement: Supplementary file 2 — Additional file 2. Internet list. [file 12913_2019_4539_MOESM2_ESM.docx]

**Additional file 2: Guideline clearinghouses and related international cancer organizations**

| **name** | **website** |
| --- | --- |
| National Guideline Clearinghouse | https://www.guideline.gov/ |
| National Institute for Health and Clinical Excellence | https://www.nice.org.uk/ |
| Scottish Intercollegiate Guidelines Network | http://www.sign.ac.uk/ |
| National Comprehensive Cancer Network | https://www.nccn.org/ |
| European Society For Medical Oncology | http://www.esmo.org/ |
| American Society of Clinical Oncology | https://www.asco.org/ |
| American Cancer Society | http://www.acsjournals.com/view/0/index.html |
| Oncology Nursing Society | https://www.ons.org/ |
| Registered Nurses’ Association of Ontario | http://rnao.ca/ |
| China Guideline Clearinghouse | http://www.cgc-chinaebm.org/index.aspx |
